# Transthyretin Stabilizers and Seeding Inhibitors as Therapies for Amyloid Transthyretin Cardiomyopathy

**DOI:** 10.3390/pharmaceutics15041129

**Published:** 2023-04-03

**Authors:** Paolo Morfino, Alberto Aimo, Giuseppe Vergaro, Chiara Sanguinetti, Vincenzo Castiglione, Maria Franzini, Marco Alfonso Perrone, Michele Emdin

**Affiliations:** 1 Interdisciplinary Center for Health Sciences, Scuola Superiore Sant’Anna, 56127 Pisa, Italy; paolomorfino12@gmail.com (P.M.); albertoaimo@libero.it (A.A.); vergarog@gmail.com (G.V.); vincenzocastiglione93@gmail.com (V.C.); emdin@ftgm.it (M.E.); 2 Cardiology Division, Fondazione Toscana Gabriele Monasterio, 56124 Pisa, Italy; 3 Department of Translational Research and New Technologies in Medicine and Surgery, University of Pisa, 56126 Pisa, Italy; chiara.sanguinetti@phd.unipi.it (C.S.); franzinimaria@gmail.com (M.F.); 4 Division of Cardiology and CardioLab, Department of Clinical Sciences and Translational Medicine, University of Rome Tor Vergata, 00133 Rome, Italy

**Keywords:** cardiac amyloidosis, transthyretin, stabilizers, seeding, tafamidis

## Abstract

Transthyretin (TTR) amyloid cardiomyopathy (ATTR-CM) is a progressive and increasingly recognized cause of heart failure which is associated with high mortality and morbidity. ATTR-CM is characterized by the misfolding of TTR monomers and their deposition within the myocardium as amyloid fibrils. The standard of care for ATTR-CM consists of TTR-stabilizing ligands, such as tafamidis, which aim at maintaining the native structure of TTR tetramers, thus preventing amyloid aggregation. However, their efficacy in advanced-staged disease and after long-term treatment is still a source of concern, suggesting the existence of other pathogenetic factors. Indeed, pre-formed fibrils present in the tissue can further accelerate amyloid aggregation in a self-propagating process known as “amyloid seeding”. The inhibition of amyloidogenesis through TTR stabilizers combined with anti-seeding peptides may represent a novel strategy with additional benefits over current therapies. Finally, the role of stabilizing ligands needs to be reassessed in view of the promising results derived from trials which have evaluated alternative strategies, such as TTR silencers and immunological amyloid disruptors.

## 1. Introduction

Systemic amyloidosis is a heterogeneous group of disorders characterized by the deposition of misfolded proteins that form cross-β sheet-rich amyloid fibrils within the extracellular space of several organs, including the heart [1]. Cardiac amyloidosis represents the leading cause of restrictive cardiomyopathy, which is an infiltrative disease associated with severe morbidity and mortality [2,3]. Although there are more than 30 identified amyloidogenic proteins, almost all cases of cardiac amyloidosis derive from aggregated transthyretin (TTR) or immunoglobulin light-chains [4]. TTR is a circulating tetrameric protein, mainly recognized as a transporter of thyroxine ($T_{4}$) and retinol. TTR amyloidosis (ATTR) may be acquired or hereditary. In the first case, it is due to wild-type TTR (ATTRwt), and in the second, it is also known as variant amyloidosis (ATTRv) and can be triggered by more than 130 pathogenic mutations. ATTR-related cardiomyopathy (ATTR-CM) is an increasingly recognized cause of heart failure (HF) in older adults, with an estimated prevalence of ≅15% among patients with HF and preserved ejection fraction [5].

The growing interest in the scientific community has led to significant advances in diagnostic and therapeutic strategies that have radically changed the epidemiology of the disease, now no longer considered rare and incurable [6].

The development of disease-modifying drugs for ATTR-CM has, therefore, become a crucial goal [7]. Disease-modifying therapy acts on different steps of amyloidogenesis with the aim of changing the natural history of the disease. Some of the proposed approaches to treat ATTR amyloidosis include blocking TTR synthesis in the liver, stabilizing TTR tetramers, disrupting TTR fibrils, and inhibiting amyloid aggregation. To date, tafamidis, which is a TTR tetramer stabilizer, is the only agent approved for the treatment of ATTR-CM. Treatment with tafamidis significantly improves the prognoses of patients, but its limited efficacy in the advanced stages of disease or after long-term treatment is still a source of concern, suggesting the existence of other pathogenetic factors involved in the disease’s progression. Another TTR stabilizer under evaluation is acoramidis (NCT03860935; NCT04622046).

Many studies and much clinical evidence indicate that amyloidogenesis proceeds via a nucleation-dependent pathway which strongly depends on concentration of amyloid fibril fragments, called “seeds” [8]. In several diseases, including ATTR-CM, misfolded proteins aggregate into seeds that promote the unfolding of native proteins, causing them to aggregate and form pathogenic assemblies ranging from low- to high-molecular-weight oligomers [9]. The mechanism of amyloid deposition in tissues, called “amyloid seeding”, has been extensively studied in vitro, but only poorly in vivo. From these studies, it emerged that “seeding” is a mechanism of amplification and diffusion of amyloid aggregates which can be enhanced and accelerated by the presence of truncated pre-formed fibrils.

The use of structure-based inhibitors against amyloid TTR seeds (e.g., TabFH2), aiming at blocking the nuclei of pre-formed ATTR fibrils in tissues, may be a complementary strategy to current therapies.

This literature review attempts to summarize the main evidence on the current role of TTR stabilizers and seeding inhibitor peptides in the setting of ATTR-CM. Herein, we discuss their possible combinations and future perspectives in light of the promising results being derived from other therapeutic strategies.

## 2. The Amyloidogenic Cascade: From TTR Tetramer to Amyloid Propagation

### 2.1. TTR: Structure and Function

TTR is a transport protein mainly circulating in the plasma and cerebrospinal fluid, and is mostly synthesized by the liver and choroid plexus. The main function of TTR is transporting thyroid hormones, especially $T_{4}$ and retinol (vitamin A), through the retinol-binding protein [10].

**Figure 1:**
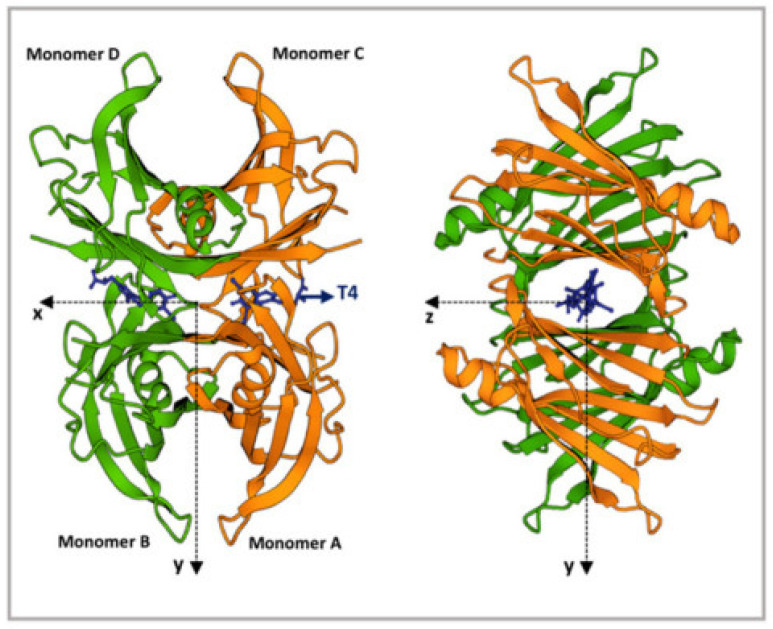
TTR structure. Ribbon diagram of the TTR tetramers. The X-axis passes through the T4 binding channels, which are formed at the interface of monomers D-B and C-A. T4 is represented in blue. The figure was produced using the “www.rcsb.org” website (protein ID code 1QAB), access date 22 June 2022. Modified with permission from C. Sanguinetti et al., 2022 [14].

TTR is characterized by a tetrameric structure with a central hydrophobic channel placed between the dimers (Figure 1) [11]. This channel contains the binding site for $T_{4}$, interaction with which enhances the structural stability of the tetramer [12]. Many stabilizing ligands (e.g., tafamidis and diflunisal) are based on this principle. TTR monomers are rich in β-strands and show an intrinsic propensity to aggregate into amyloid fibrils [13].

### 2.2. Why Does TTR Tetramer Dissociate? What Are the Consequences?

In vitro studies suggest that the native tetramer dissociation is the rate-limiting step, as well as the starting point, of TTR amyloidogenesis [15]. Tetramer dissociation is a complex process with multiple intermediates, and the disruption of the native tetramer is a limiting step in both TTRwt and TTRv, since it shows the highest energy barrier of the transition state [16]. Current knowledge states that the tetramer first dissociates to dimers, and then the dimers dissociate to monomers. Partial monomer unfolding, likely due to β-strand dissociation, causes the formation of amorphus oligomers and their subsequent deposition as amyloid fibrils according to the thermodynamically favorable downhill process [17,18]. Therefore, tetramer stabilization should prevent the dissociation of TTR and subsequent fibril aggregation [19]. Tetramer dissociation is likely a multifactorial process, with many promoting factors identified under laboratory conditions (e.g., concentration, temperature, pH) but still not elucidated in vivo [20,21]. Factors that are likely to be involved in the pathogenesis of ATTR by promoting TTR instability mainly include age-related failure of proteostasis, destabilizing TTR mutations, and oxidative modifications [21]. Indeed, the more severe and earlier-onset forms of the disease occur due to some forms of ATTRv associated with highly pathogenic TTR variants [22]. On the other side, protective *TTR* mutations and stabilizers shift the equilibrium towards the folded tetramer.

Based on recent research, in vivo fragmentation of the precursor proteins by endogenous proteases represents one of the most prominent aspects involved in both amyloid fibril formation and fragmentation. Further studies have reported that a proteolytic cleavage in the CD loop dramatically destabilizes the tetramer, suggesting that the mechano-enzymatic cleavage may lead to aggregation of the C-terminal fragment (residue 49-127) in vivo [23,24]. Proteolysis causes the loss of monomers secondary structures, thus promoting the destabilization of tetramers and the release of the 49-127 C-terminal peptides that, indeed, have been identified in in vivo TTR amyloid fibrils [19,25].

The proteases responsible for TTR cleavage have not yet been identified, but the fragmentation pattern suggests the involvement of trypsin-like proteases, fibrinolytic agents (e.g., plasmin), and subtilisin [26,27,28,29]. Indeed, patients with ATTR amyloidosis clinically manifest higher plasma proteolytic activity [30].

### 2.3. Amyloid Formation and Tissue Deposition

Although several amyloidogenic proteins exist, the mechanism of fibril and plaque deposition is common because it is driven by the peculiar structure, rich in β-sheets, and by the propensity to misfold. These proteins display short sequences, known as amyloidogenic regions, which play a key role as nucleating centers in forming the common spines of fibrillar aggregates by increasing the number of β-sheet structures [31]. These aggregation-prone regions are sequences of few amino acids and are mostly hidden within the native proteins, where they constitute the hydrophobic core-stabilizing tertiary structure [32]. Self-association of the two TTR β-strands F and H, which are normally buried in the tetramer (between the dimers interface) and exposed in monomers, seems to drive amyloid fibril formation [33]. The contributions of other aggregation-prone segments to the amyloid core are not excluded.

**Figure 2:**
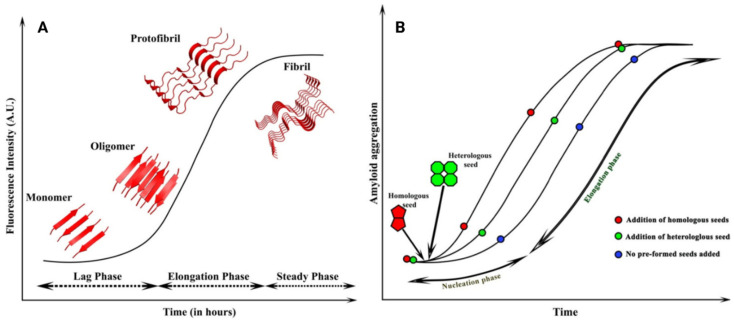
Schematic representation of amyloidogenic cascade and amyloid seeding. (**A**) The sigmoidal curves exhibit different forms because monomers, oligomers, protofibrils, and fibrils are formed at various phases depending on time. (**B**) Amyloid seeding: the addition of pre-formed seeds reduces the lag phase, leading to faster aggregation. Homologous seeds, which have the same nature as the existing nuclei, lead to homologous seeding, whereas the heterologous seeds differ from the initial nuclei and lead to heterologous or cross-seeding. Modified with permission from Subedi S. et al., 2022 [36].

Amyloid formation can be described by kinetics involving a lag phase (nucleation), an elongation phase (growth), and a plateau phase (steady) (Figure 2A). Initially, the oligomerization step implicates critical nucleus development, which is the most kinetically unstable species [8]. This process occurs during the lag phase and is termed as primary nucleation [34]. The nucleus is the smallest amyloid precursor and provides support for subsequent fibril growth. Oligomer formation occurs contextually, but only a certain amount of them enter the fibril growth pathway [35]. The lag phase duration may be dramatically reduced by adding pre-existing seeds.

Then, the elongation step occurs, which is the addition of misfolded monomers to the unstable nucleus. This aggregation is spontaneous because of the reduction in free energy caused by the formation of chemical bonds, which stabilize the compound [37]. The process continues until the amyloid fibril reaches a plateau and a lower intrinsic energy, thus exhibiting a characteristic sigmoidal curve. However, once fibrils are generated, they can dynamically fragment and release small terminations that induce the recruitment of other monomers and the generation of new fibrils, acting as nuclei themselves. Fragmentation of amyloid fibrils in vivo may be enhanced by endogenous proteases [26].

Amyloidogenesis is very slow and might require years to spontaneously occur in vitro, suggesting that the formation of fibrils in vivo may require the existence of catalyzing factors. Finally, amyloid fibrils may also assemble with each other and with other compounds, such as the serum amyloid protein or extracellular components, to form amyloid plaques. These then infiltrate the extracellular environment, causing tissue and cellular damage [8]. Whether amyloid deposits directly induce organ dysfunction or represent epiphenomena is still unclear. Progressive amyloid deposition in the heart is the main mechanism of damage in ATTR-CM amyloidosis, whereas the toxic effect of TTR oligomeric species seems to be less relevant.

These differences are corroborated by the clinical observation of a grater amyloid burden in the heart with relatively preserved ejection fraction in ATTR-CA compared to a smaller amyloid burden, but also by severe systolic dysfunction, as observed in light chain amyloidosis [38].

### 2.4. Structure of Amyloid Fibrils

**Figure 3:**
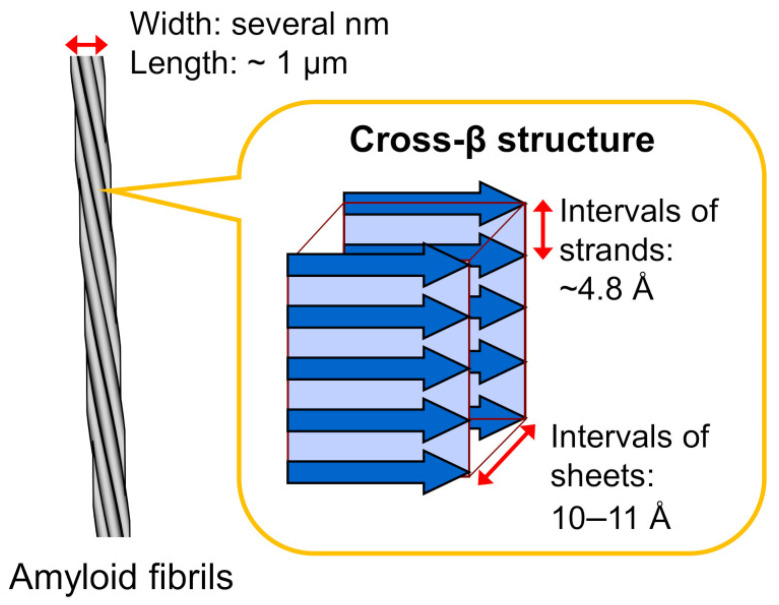
Schematic illustration of an amyloid fibril. Amyloid fibrils typically show unbranched morphology, which consists of several laterally bundled protofilaments a few nanometers in width and around a micrometer in length. Protofilaments show a peculiar cross-β structure, where β-strands are stacked perpendicular to the long axis of the fibril. Reprinted with permission from Chatani E. et al., 2021 [41].

Amyloid fibrils are 5–15 nm in width and several micrometers in length. They are insoluble fibrous structures derived from the polymerization of hundreds to thousands of monomeric peptides. This process requires the assembly of a momentarily or permanently misfolded protein into pre-fibrillar species. These heterogeneous and potentially cytotoxic intermediates subsequently combine to form higher-order structures, namely, protofilaments [8]. The fundamental structure of all amyloid substances is the fibril, which consists of twisted protofilaments. Amyloid fibrils also show a peculiar structure, called “cross-β”, which derives from the lamination of consecutive β-sheet layers [39]. This organization resembles a ladder of β-strands placed orthogonally to the fibril axis, with each rung separated from the others by a space that presents a hydrogen bond (Figure 3) [8]. Each amyloid fibril displays a unique dry steric zipper which represents the stabilizing core of the fibril [40]. The result is a very stable structure.

TTR fibrils contain a number of protofilaments, ranging from one to five [42]. It has been shown that TTRwt tends to form linear oligomers, while TTRv tends to form different tangles [43]. Furthermore, several studies have suggested the existence of two different types of TTR fibrils: type A amyloid fibrils, formed by a mixture of both cleaved C-terminal and full-length TTR, and type B amyloid fibrils, composed only of full-length TTR [44]. Patients with type A fibrils within the myocardium show worse prognoses, probably due to a higher concentration of truncated species triggering amyloid seeding [45].

### 2.5. Promotion and Further Deposition: Amyloid Seeding

Amyloid seeds are small, fibrillary fragments that are capable of inducing native proteins to assume similar pathogenic and insoluble structures. The concept of seeds derives from studies on prion diseases (e.g., Creutzfeldt–Jakob disease, Kuru, fatal insomnia), which share many pathological features with amyloidosis [46]. Saelices and colleagues demonstrated that seeds obtained from ex vivo ATTR tissues caused fibril formation of either TTRwt or TTRv in vitro through a seeded-nucleation process [47]. However, Ranlov and co-workers had already recognized the pathogenic meaning of seeding in 1960s. Later, Kisilevsky and co-workers described the “amyloid enhancing factor” as a transmissible factor which promotes amyloid deposition [48,49].

Amyloidogenesis is a process that self-propagates through the formation and spread of new seeds, which derive from the spontaneous and proteolysis-mediated fragmentation of pre-formed fibrils [50]. The rate-determining step in the formation of amyloid fibrils is the generation of the nuclei during the lag phase [51]. Therefore, the initial slow phase may be shortened or even abolished by the addition of pre-formed seeds, which rapidly trigger protein aggregation into fibrils, thus bypassing the lag phase (Figure 2B) [9,52]. This process is referred as “amyloid seeding” and can be promoted by both homologous and heterologous fibril fragments [36]. During the process, the unstructured monomers are converted into semi-structured seeds and, finally, into amyloid fibrils. The surfaces of amyloid seeds contain active sites at the ends of which growth by elongation takes place. Seeds also contain surfaces parallel to the cross-β hydrogen bonds that have been proven to catalyze nucleation of new amyloids (secondary nucleation) [53]. Since each fibril generates novel seeds, we may assume that the greater the amyloid tissue content, the faster further amyloid deposition progresses. Therefore, if amyloid fibrils remain in the tissues, they are expected to produce seeds which convert TTR, thus perpetuating the disease independently of the administration of other therapies.

ATTR disease progression after liver transplantation, as well as the correlation between the clinical course of the disease and the kinetics of amyloid formation, represent indirect evidence of the presence of amyloid seeds in vivo [54]. On the other hand, tissue visualization through electron microscopy provides direct proof of the presence of amyloid seeds [55].

ATTR seeds do not induce amyloid aggregation of TTRwt under physiological conditions (pH = 7.4). However, seeds extracted from ATTRv D38A cardiac tissue promote amyloid formation from both the TTRwt and TTRv tetramers under acidic environments (pH = 4.3), since acidic pH helps to obtain the TTR tetramer dissociation. The addition of ex vivo seeds also induces the aggregation of misfolded TTR monomers in a dose-dependent manner [47]. Furthermore, the ATTR seeds obtained through sonication, which disrupts physical bonds between the proteins, showed great enhancement in their seeding capacity because of their higher concentration of fibril fragments [47,56].

An analysis conducted on an engineered monomeric TTRv which carried a double mutation, F87M-L110M, reported that ex vivo ATTR seeds accelerated the amyloid formation of TTR monomerized by mutations under physiological conditions (pH = 7.4); it normally exposes the adhesive amyloidogenic segment, contrarily to TTRwt [57]. These results, obtained in vitro, suggest that amyloid seeding only occurs after the dissociation of the tetramer into monomers. The injection of seeds possibly does not normally induce amyloid formation in vivo because it previously needs the TTR tetramer dissociation [58,59]. Moreover, seeded fibrils can also further accelerate the formation of TTRwt or TTRv fibrils, thus acting as seeds themselves [47].

Other analyses revealed that amyloid seeding does not correlate with age, gender, genotype, pathology, or tissue type. However, the seeding capacity is strongly associated with the concentration of C-terminal fragments, which are abundantly detected in patients with type A amyloid fibrils [47]. This connection even explains the higher risk of amyloid deposition in patients with the A phenotype [45]. Despite the stabilization that occurs through tafamidis and diflunisal, amyloid seeds have been demonstrated to promote the deposition of TTRwt into amyloid fibrils in the presence of the stabilizers at concentrations that fully inhibit the aggregation of TTR when no seeds are present [60].

## 3. TTR Stabilizers

Until the late 1990s, treatment options for ATTR-CM were limited to supportive therapies. A minority of patients received heart transplant, which is mostly recommended among young subjects with ATTRv (mainly V30M) showing early-stage disease and limited extracardiac involvement [4,61]. Stabilization of the TTR tetramer has become an important target for novel therapies to halt the progression of ATTR amyloidosis. TTR stabilizers prevent tetramers from dissociating by binding to the $T_{4}$-binding site on TTR (e.g., tafamidis and diflunisal) or by reproducing the structural stabilizing effect of *TTR* variant T119M (e.g., acoramidis) [4,62]. Other compounds with some stabilizing effects have been evaluated, namely, tolcapone and epigallocatechin-3-gallate (EGCG).

### 3.1. Tafamidis

Tafamidis is an orally bioavailable benzoxazole derivative that inhibits the dissociation of both TTRwt and TTRv tetramers by binding the $T_{4}$-binding site [63]. Tafamidis was first approved for the treatment of adults with ATTRv-related familial polyneuropathy (ATTR-FAP). Tafamidis was the first disease-modifying therapy to receive approval for use in the treatment of adults with both -wt and -v ATTR-CM in many countries, including the USA, Japan, and part of the European Union [64,65,66].

In the phase III trial ATTR-ACT study, which involved 441 patients with ATTR-CM (ATTRwt and ATTRv in 76% and 24%, respectively), patients were randomized in a 2:1:2 ratio to receive tafamidis 80 mg or 20 mg or a placebo for 30 months. Most patients were male (90%) and had a median age of 75 years at baseline. The treatment with tafamidis was associated with lower all-cause mortality than in the placebo group (78 of 264 (29.5%) vs. 76 of 177 (42.9%); hazard ratio (HR): 0.70, 95% confidence interval (CI): 0.51–0.96), and a lower rate of cardiovascular hospitalizations (relative risk ratio (RR): 0.68 (0.48 per year vs. 0.70 per year; 95% CI 0.56–0.81)) [67]. The Kaplan–Meier survival curves for all-cause mortality diverged 18 months after the first dose. The administration of tafamidis also had beneficial effects on symptoms, as demonstrated by the lower rate of decline in 6 min walking distance (6MWT), and quality of life, as measured through the Kansas City Cardiomyopathy Questionnaire Overall Summary (both *p* < 0.001), with differences first observed after 6 months. The drug was also well tolerated, without onset of significant adverse events compared to the placebo. Interestingly, tafamidis did not demonstrate any beneficial effects in terms of cardiovascular hospitalizations in patients with advanced-stage disease (New York Heart Association (NYHA) class III), probably because of the underpowered subgroup analysis attributable to longer survival during more severe periods of disease [67]. Further analysis showed that the effect of tafamidis is dose-dependent. Although both 80 and 20 mg doses stabilized the tetramer in a significant proportion of patients, only the higher one reached the optimal plateau of stabilization. Moreover, patients administered 80 mg had higher mean TTR concentrations compared with those administered under 20 mg, suggesting that a lower amount of dissociated TTR was consumed in the amyloidogenic cascade [68].

In the long-term extension study, after a median follow-up of 58.5 months, patients continuously treated with tafamidis (n = 176) had significantly better survival than those first treated with the placebo (n = 177) (HR: 0.59, 95% CI 0.44–0.79), independently of NYHA class genotype, at baseline. Mortality reductions were generally confirmed across NYHA subgroups, showing a 44% decrease in the risk of all-cause mortality in patients with NYHA class I or II (HR: 0.56, 95% CI 0.38–0.82) and a 35% decrease in patients with NYHA class III (HR: 0.65, 95% CI 0.41–1.01, *p* = 0.06) [69]. Subjects in NYHA class IV, representative of very advanced disease, were excluded from this trial. Moreover, patients switching from the placebo to the tafamidis arm reported an improvement in survival compared with the patients who continued on the placebo [69].

### 3.2. Acoramidis

Acoramidis (AG10) is a small stabilizer molecule with oral bioavailability. AG10 emulates the stabilizing property of the T119M variant, which is likely due to the development of hydrogen bonds between the residues of ${Ser}^{117}$ of TTR monomers [70]. Based on in vitro studies, the efficacy and selectivity of acoramidis appear to be superior to tafamidis and diflunisal [71].

The safety and tolerability of acoramidis was evaluated in a phase II study which enrolled 49 patients with both variant and wild-type symptomatic ATTR-CM. In comparison with the placebo group, patients taking 800 mg twice daily for 28 days showed >50% increased circulating TTR levels and a near-complete stabilization of TTR tetramers at peak and trough acoramidis serum concentrations [72]. The ATTRIBUTE-CM study is an ongoing phase III trial designed to evaluate the safety and efficacy of acoramidis. This multicenter study enrolled 510 participants and consists of 2 survey phases [73]. At the end of phase A, the treatment with acoramidis (800 mg twice a day) was associated with a reduction in serum NT-proBNP levels and an improvement in quality of life. However, this treatment did not reach the primary end-point, which was defined as an improvement in the 6MWT. Phase B, with a follow-up of 30 months, will evaluate the ability of acoramidis to reduce all-cause mortality and hospitalization for cardiovascular causes [74]. Further results are expected from a phase III study in Japanese participants with symptomatic ATTR-CM (NCT04622046) to establish its pharmacokinetic and pharmacodynamic profile among 22 patients after 30 months of follow-up [75].

### 3.3. Diflunisal and Other Anti-Inflammatory Drugs

Diflunisal is a non-steroidal anti-inflammatory drug (NSAID) synthesized from 2,4-difluoroaniline that ties the $T_{4}$-binding site similarly to tafamidis, but with lower affinity. A phase I study showed that diflunisal (250 mg twice daily) complexes to TTR, preventing amyloid fibril formation in vitro [76]. Although diflunisal has been shown to halt disease progression and improve quality of life in some retrospective analyses, most studies had a shorter duration of follow-up (12–24 months) compared to the 30 months follow-up of ATTR-ACT [76,77,78,79,80]. It was shown that diflusinal was not effective in relieving cardiac dysfunction (assessed as left ventricular [LV] mass, ejection fraction, or biomarkers) in a single-arm, open-label study [81].

It is possible that diflunisal could be administered to patients with ATTR amyloidosis and reduced access to new therapies [81,82]. Renal and gastrointestinal side effects need to be cautiously considered since the chronic inhibition of cyclo-oxygenases can exacerbate pre-existing kidney dysfunction, leading to fluid overload and arterial hypertension [70].

Flufenamic acid is a NSAID showing increased binding affinity for TTRwt and greater slowing of tetramer dissociation compared with diflunisal after in vitro acid-induced fibril formation [83]. The compound has also been demonstrated to be effective against the common familial TTR variants. Under similar conditions, the NSAID diclofenac was reported to be a worse inhibitor for fibril formation than diflunisal, making it a poor candidate for further studies [83]. In vitro and ex vivo analysis showed that some analogues of the NSAID flurbiprofen were more effective in selectively binding the TTR native structure in diluted plasma than diflunisal, but less effective in comparison with tafamidis. In addition, the authors found that limited changes within the molecule structure led to a substantial increase in the ability to stabilize TTR [84]. Finally, the administration of resveratrol, which is a natural phenol with antioxidant and anti-inflammatory effects, resulted in TTR tetramer stabilization similar to diflunisal, increasing the protein plasma levels in transgenic mice carrying one copy of the TTR gene [85].

### 3.4. Tolcapone and ECGC

Tolcapone is a catechol-O-methyltransferase inhibitor traditionally used for the treatment of Parkinson’s disease [86]. Its stabilizing action on TTR may be due to a high affinity for the $T_{4}$-binding site. Tolcapone has shown fibril disruption activity in vitro, suggesting a potential for the regression of amyloid and disease. In vitro, tolcapone binds with high affinity and inhibits the aggregation of TTRwt and TTR variant V122I [87]. A phase IIa proof of concept clinical trial which enrolled 17 subjects with ATTR (cardiac involvement in 55% of cases) found that tolcapone was associated with an increase in TTR stabilization in plasma after 15 weeks of follow-up [88]. However, the role of tolcapone in ATTR was not further investigated.

EGCG is a green tea catechin that is able to inhibit the formation of amyloid fibrils by promoting tetramer stabilization [89]. EGCG has been shown to bind and disrupt various amyloid deposits into smaller, non-toxic aggregates [90,91]. The efficacy of EGCG was tested in a single-center study in which 19 ATTR-CM patients took EGCG daily for 12 months. The patients reported no disease progression, a reduction in mean LV myocardial mass (12.5%), and an increase in mean mitral annular velocity (9%) [92]. Other single-arm studies confirmed the modest changes in cardiac parameters [93]. EGCG was well tolerated in a real-world cohort of ATTR-CM patients (n = 65), but it was not associated with an improvement in survival after a median follow-up of 691 days [94].

## 4. Seeding Inhibitors

Seeding inhibitor therapies are emerging treatments which aim to halt amyloid deposition by blocking amyloid seeds. TabFH2 is a novel peptide inhibitor designed to selectively bind and halt amyloidogenic segments of TTR seeds [70].

### TabFH2

The primary seeding inhibitor under development is represented by a structure-based peptide inhibitor designed and optimized to cap the tips of TTR fibrils involved in amyloid aggregation. Indeed, these peptide inhibitors halt amyloid seeding in vitro by targeting the F and H β-strands of TTR, which have been identified as driving-aggregation segments primarily leading to seed formation [33].

The current most effective seeding inhibitor is termed as “Tab” (from “TTR aggregation blockers”), followed by the letters F and H, which represent the target strands, and by the number 2, which refers to the second optimized version of the peptide [47]. The mixture of the peptides TabF2 and TabH2 (i.e., TabFH2) has been shown to efficiently inhibit the amyloidogenic seeding of TTR [60]. Peptide systems have already been employed as potential inhibitors of protein aggregation in several conditions involving pathological protein aggregation, for example, a peptide inhibitor of p53 aggregation has been utilized to prevent some types of ovarian cancer, and inhibitors of tau aggregation have been applied in the setting of Alzheimer’s disease [95,96]. Furthermore, recent studies have reported that the inhibition of TTR amyloid seeding and aggregation may occur using monoclonal antibodies (mAbs), which target the amyloidogenic segments [97]

**Figure 4:**
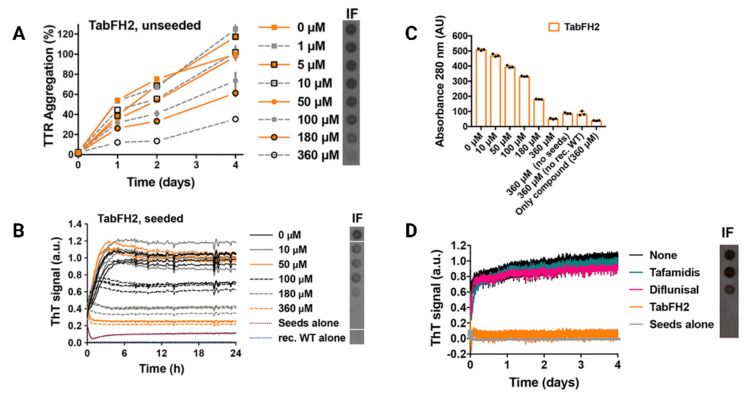
TabFH2 inhibits TTR aggregation and amyloid seeding caused by ATTR ex vivo seeds. (**A**) Inhibition of TTR aggregation by TabFH2 in the absence of seeds, measured by absorbance at 400 nm. Increasing amounts of TabFH2 were added to 1 mg/mL of recombinant WT TTR, and the sample was incubated for 4 days at pH 4.3. Absorbance measured after 4 days of incubation in the absence of TabFH2 was considered 100% aggregation, because no soluble TTR was detected (n = 3). Error bars, S.D. Right inset, anti-TTR dot-blot of insoluble fractions (IF) collected by centrifugation after 4 days of incubation. (**B**) Inhibition of amyloid seeding by TabFH2 at pH 4.3, monitored by ThT fluorescence. Increasing amounts of TabFH2 were added to 0.5 mg/mL of recombinant WT TTR and 30 ng/L of ATTR-D38A seeds. All replicates are shown (n = 4). a.u., arbitrary units. Inset, anti-TTR dot-blot of IF collected by centrifugation after 24 h of incubation. All samples were spotted onto the same nitrocellulose membrane and subjected to the same procedure; splicing was needed for presentation purposes. (**C**) Protein content quantification of the insoluble fractions collected from B, measured by 280 nm absorbance. AU, absorbance units. The reduction in ThT fluorescence observed in B correlates with the decrease in total protein and TTR content in the IF, shown in the right inset of B. (**D**) Comparison of inhibition of amyloid seeding by tafamidis, diflunisal, and TabFH2 when incubated for 4 days, measured by ThT fluorescence. A 360 M inhibitor was added to 0.5 mg/mL of recombinant WT TTR and 30 ng/L of ATTR-D38A seeds (n = 3). Error bars, S.D. Inset, anti-TTR dot blot of IF collected by centrifugation after 4 days of incubation. Modified with permission from L. Saelices et al., 2019 [60].

In vitro studies have reported that TabFH2 completely halts TTR aggregation seeded by ex vivo fibrils in a dose-dependent manner, with enhanced efficacy at higher doses (Figure 4) [60]. The inhibition mechanism requires that TabFH2 bind to the ATTR seeds. Further results revealed that TabFH2 optimally inhibits amyloid seeding induced by both TTRwt and by six variants of TTRv ex vivo fibrils in a tissue-independent manner, resulting in a decrease in amyloid conversion. Moreover, since the amyloid seeding correlates with the concentration of TTR seeds, TabFH2 showed an inverse correlation with them. In the same study, the authors demonstrated that tafamidis and diflunisal were not able to prevent amyloid formation in the presence of seeds, differently from TabFH2 [60]. This concept may be crucial for patients with ATTR-CM, who often receive a diagnosis when they manifest advanced TTR deposition and have limited treatment options.

**Figure 5:**
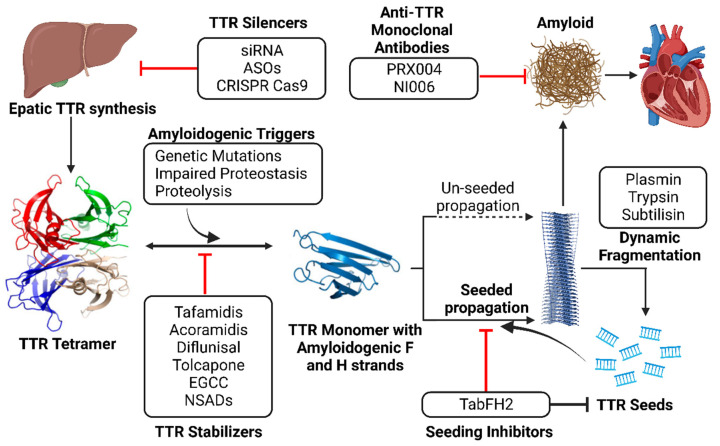
Multi-target approach to ATTR amyloidosis. TTR dissociation provides monomers with high propensity to nucleation and fibril formation through self-association of amyloid-driving segments. TTR stabilizers maintain the tetrameric form, halting tetramer dissociation and unseeded polymerization. After spontaneous and proteolytic fragmentation of fibrils, small fragments may serve as seeds that induce amyloid seeded propagation. The peptide inhibitor TabFH2 does not affect tetramer stability, but it binds to seeds, hindering self-recognition and seeding. Both strategies seem synergistic and could potentially be used in combination. The contemporary administration of TTR silencers and anti-TTR monoclonal antibodies may prove effective since they block TTR synthesis and promote amyloid regression.

The effects of TabFH2 were tested in two *Drosophila* models of ATTR with neurologic involvement. The study reported that TabFH2 decreased amyloid TTR deposition within the thoracic adipose tissue and brain glia, but also prevented the locomotor impairment compared with diflunisal, resulting in the improvement of motor parameters compared with the control group of treated engineered flies expressing the less severe double mutation pV14N/pV16E (mean velocity: 3.8 mm/s vs. 2.7 mm/s, *p* = 0.029; total distance travelled: 262 mm vs 143 mm, *p* = 0.005, after 17 days of treatment) [98]. These initial results deriving from in vivo studies encouraged the development of promising peptide seeding-inhibitors to delay ATTR progression and the possible use of combined therapies with TTR stabilizers (Figure 5).

## 5. Which Perspectives for TTR Stabilizers and Seeding Inhibitors?

### 5.1. TTR Stabilizers: Will They Survive to the Introduction of TTR Silencers Possibly Coupled to mAbs Clearing Tissue Amyloid?

Besides TTR stabilizers, the second choice for treating ATTR amyloidosis involves the use of TTR gene-silencing agents. These molecules inhibit TTR gene expression within hepatocytes, thus reducing the production of the circulating amyloid precursor [99]. TTR gene silencers consist of small interfering RNAs (siRNA), such as patisiran, revusiran, and vutrisiran; antisense oligonucleotides (ASOs), such as inotersen and eplontersen; and the novel gene editing technology represented by CRISPR Cas9 [4,100]. Both siRNA and ASOs selectively bind to complementary mRNA molecules and promote their degradation to prevent TTR expression. CRISPR Cas9 is a promising genomic editing strategy based on the Cas9 nuclease, which is bound to a guide RNA capable of irreversibly silencing the target gene [101].

For ATTR-FAP patients, the FDA has recently approved the siRNA patisiran and the ASO inotersen as treatments, regardless of the presence and degree of ATTR-CM. Interestingly, patisiran improves the cardiac phenotype in patients with ATTR-FAP and concomitant ATTR-CM [102]. The ability of patisiran to promote the recovery of LV structure and function is currently under investigation with tafamidis. However, the effect of patisiran on participants with ATTR-CM who have never taken other disease-modifying therapies will be evaluated in the ongoing phase III trial APOLLO-B (NCT03997383) [103]. Furthermore, the ongoing phase III clinical trials will evaluate the siRNA vutrisiran (HELIOS-B, NCT04153149) and the novel ASO eplontersen (CARDIOTTRansform, NCT04136171) in patients with ATTR-CM [104,105].

Despite the proven ability to stabilize or silence TTR, none of the currently approved therapies significantly affect pre-existing amyloid fibrils. In the ATTR-ACT study, patients did not manifest significant reductions in LV wall thickness compared with the placebo group after 30 months [67]. Similarly, in a general study population, patisiran was not able to reduce LV wall thickness compared with the placebo [102,106]. Therefore, the tissue regression of amyloid deposits through chemical degradation (EGCG, doxycycline, and tauroursodeoxycholic acid) or immune-mediated amyloidolysis represents a critical goal of the current research. The evaluation of the anti-TTT mAb PRX004 was the object of a phase I study (NCT03336580) that was interrupted before term due to the COVID-19 pandemic. Collected data from 7 ATTRv-CM patients showed an improvement in neurological symptoms and heart function after 9 months of treatment [107]. The safety and tolerability of this drug are under evaluation in an open-label phase I study including ATTRv patients [108].

Other anti-TTR mAbs include Ab-A and NI006, which have shown affinity for binding both TTRv and TTRwt, inducing the regression of amyloid deposits both in vitro and in vivo [109,110]. A phase I study on NI006 (NCT04360434) in patients with ATTR-CM is underway [111].

TTR targeted gene therapy and TTR tetramer stabilizers could, hypothetically, be combined. Although tafamidis is the first disease-modifying therapy specifically approved for the treatment of ATTR-CM, it is an expensive drug which needs long-term administration before a clinical response is given. Therefore, a clinical trial evaluating this approach is unlikely, as is its use in daily clinical practice, since the association would likely not be cost-effective [112]. In conclusion, the promising results from trials evaluating TTR silencers and immunological amyloid disruptors might limit, in the future, the role of TTR stabilizers in ATTR-CM.

### 5.2. Seeding Inhibitors: Will They Ever Become a Viable Therapeutic Option?

Amyloid seeding strongly promotes further TTR deposition in vitro, and it possibly contributes to disease progression in vivo, especially in the advanced stages of disease when there is a large concentration of pre-formed fibrils. Although recent data on long-term outcomes of tafamidis are partly in contrast with the following concept, it is plausible to consider seeding as a cause of the limited efficacy of current therapies [69]. Therefore, seeding inhibitors may play a crucial role if administered with complementary therapies, such as TTR stabilizers, in the treatment of ATTR amyloidosis and related-CM. Based on the kinetics of seeded propagation, anti-seeding peptides could be greatly effective if used before the curve of deposition dramatically changes his inclination, thus preventing a rapid exacerbation of clinical symptoms.

Since the role of anti-seeding therapies aims at blocking faster amyloid aggregation without impairing pre-existing fibrils, which are, however, a source of seeds themselves, we can assume that their use with mAbs or other amyloid disruptors would be more beneficial than the combination with only TTR stabilizers. Nevertheless, current evidence suggests that patients with very early disease stages, where the amount of tissue amyloid is quite limited (e.g., carriers of TTR mutations), may manifest relief after the administration of stabilizers and seeding inhibitors. In this particular subgroup, combined treatment might dramatically delay the disease progression due to the scarce amount of amyloidogenic precursor, which would be totally inhibited.

To date, the use of seeding inhibitor is not a near future perspective. Although many preclinical studies expanded the knowledge about the pathogenesis of amyloid propagation, there are still major differences between in vitro and in vivo models, primarily due to the difficult reproducibility of ATTR-CM in experimental models, complicated by our limited understanding of its multifactorial manifestations, such as inflammation and crosstalk between different pathologies [113,114].

## 6. Conclusions

Despite the remarkable advances in the treatment of ATTR-CM, current approved therapies are only focused on stabilizing the source of the amyloidogenic TTR, and displays limited efficacy in advanced-stage disease or after long-time treatment. No approved strategy aims at depleting or blocking pre-existing amyloid fibrils, which can continue to spread and cause organ damage. In this context, anti-seeding peptides, combined with TTR stabilizers, might delay disease progression by halting amyloid seeded propagation. Further pre-clinical studies are warranted to assess the efficacy and feasibility of this approach as well as to obtain optimized structure-based seeding inhibitors. Other promising approaches include TTR silencers and immunological amyloid disruptors, which might prove even more effective than stabilizers, since they block TTR synthesis and promote amyloid regression.
